# Emerging Role of Plant-Based Bioactive Compounds as Therapeutics in Parkinson’s Disease

**DOI:** 10.3390/molecules28227588

**Published:** 2023-11-14

**Authors:** Nitu Kumari, Santosh Anand, Kamal Shah, Nagendra Singh Chauhan, Neeraj K. Sethiya, Manmohan Singhal

**Affiliations:** 1 Department of Biotechnology, School of Applied Sciences, REVA University, Bengaluru 560064, Karnataka, India; nitumicro225@gmail.com; 2 Institute of Pharmaceutical Research, GLA University, Mathura 281406, Uttar Pradesh, India; kamal0603@gmail.com; 3 Drug Testing Laboratory Avam Anusandhan Kendra, Raipur 492010, Chhattisgarh, India; chauhan.nagendra@gmail.com; 4 Faculty of Pharmacy, School of Pharmaceutical and Populations Health Informatics, DIT University, Dehradun 248009, Uttarakhand, India; manmohan.singhal@dituniversity.edu.in

**Keywords:** phytocompounds, Parkinson’s disease, phenolic acids, antiparkinsonian, neuroprotection

## Abstract

Neurological ailments, including stroke, Alzheimer’s disease (AD), epilepsy, Parkinson’s disease (PD), and other related diseases, have affected around 1 billion people globally to date. PD stands second among the common neurodegenerative diseases caused as a result of dopaminergic neuron loss in the midbrain’s substantia nigra regions. It affects cognitive and motor activities, resulting in tremors during rest, slow movement, and muscle stiffness. There are various traditional approaches for the management of PD, but they provide only symptomatic relief. Thus, a survey for finding new biomolecules or substances exhibiting the therapeutic potential to patients with PD is the main focus of present-day research. Medicinal plants, herbal formulations, and natural bioactive molecules have been gaining much more attention in recent years as synthetic molecules orchestrate a number of undesired effects. Several in vitro, in vivo, and in silico studies in the recent past have demonstrated the therapeutic potential of medicinal plants, herbal formulations, and plant-based bioactives. Among the plant-based bioactives, polyphenols, terpenes, and alkaloids are of particular interest due to their potent anti-inflammatory, antioxidant, and brain-health-promoting properties. Further, there are no concise, elaborated articles comprising updated mechanism-of-action-based reviews of the published literature on potent, recently investigated (2019–2023) medicinal plants, herbal formulations, and plant based-bioactive molecules, including polyphenols, terpenes, and alkaloids, as a method for the management of PD. Therefore, we designed the current review to provide an illustration of the efficacious role of various medicinal plants, herbal formulations, and bioactives (polyphenols, terpenes, and alkaloids) that can become potential therapeutics against PD with greater specificity, target approachability, bioavailability, and safety to the host. This information can be further utilized in the future to develop several value-added formulations and nutraceutical products to achieve the desired safety and efficacy for the management of PD.

## 1. Introduction

The brain is the most complicated organ of the body consisting of a complex network of neurons, and functions as a site of intelligence, memory, and cognition, the initiator of body movement, the interpreter of the senses, and the manager of behaviors. It mainly consists of billions of nerves, which are in regular communication through trillions of connections called synapses [1,2]. The brain is subjected to various forms of stresses, including oxidative stress (OS) resulting from the body’s oxygen requirements/utilization and high content of unsaturated fatty acids [3,4]. Nerve cells in the mid-brain degrade slowly, leading to movement- and coordination-related problems, ultimately resulting in neuropathophysiology. Neurodegeneration is associated with the progressive damage of neuronal tissue that causes the irrecoverable loss of neuronal function, subsequent decline in cognitive function, and motor activity [5,6,7]. Among the neurodegenerative diseases, Parkinson’s disease (PD), mild cognitive impairment (MCI), Alzheimer’s disease (AD), epilepsy, multiple sclerosis (MS), amyotrophic lateral sclerosis (ALS), and Huntington’s disease (HD) are of primary importance with distinguished pathological etiologies and clinical management [8,9].

PD is the second common chronic neurodegenerative disorder after AD seen mostly in the aging population [10,11]. It is characterized by synucleipathy, wherein neurons in specific part of the brain undergo damage, resulting in motor signs of muscle stiffness, tremor, and postural instability. Additionally, synucleipathy more specifically refers to a class of neurodegenerative disorders that are characterized by a prion-like spread through interconnected neuronal networks and the abnormal aggregation of α-syn inside glial or neuronal cells. However, the complete etiologies of PD still remain unclear [12,13,14]. The disease’s progression has been pointed out as being related to the depletion of dopamine in the nigrostriatal pathway. Further, intracytoplasmic inclusions of dopamine called Lewy bodies have been identified in patients with PD [12,13]. The deterioration of dopamine-producing neurons inside the substantia nigra (SN), with reduced dopamine release in the striatum, is a major cause of the disease. The SN is located in the midbrain, posterior to the crus cerebri fibers of the cerebral peduncle. It consists of two regions: the substantia nigra pars compacta (SNpc), which harbors dopaminergic neurons, and the substantia nigra pars reticulata (SNpr), comprising gamma-aminobutyric acid-responsive (or GABAergic) neuronal cells. The putamen, striatum, and caudate nuclei are well connected with dopaminergic projections from the SNpc. Each side of an adult SNpc has 400–500 thousand dopaminergic cells, constituting a negligible fraction of total brain mass. As evident in PD, these tiny clusters of cells have a disproportionate impact on motor output and behavior. When compared to other neurons, dopaminergic neurons in the SNpc are more vulnerable to oxidative stress. In the ventral midbrain, these neurons extend to the striatum and play a crucial role in regulating motor behavior in mammals. The SNpc comprises a cluster of cells that releases the neurotransmitter dopamine in the striatum connected to the basal ganglia. The basal ganglion in turn is linked to the thalamus and motor cortex, which are associated with controlling motor output. PD is predominantly attributed to the loss of the majority of cells in the SNpc region [15,16,17]. The factors that are involved in the progression of PD includes ROS, neuroinflammation, mitochondrial dysfunction, the misfolding of proteins, and protein agglomeration, along with several other environmental factors and biological processes [11]. 

Many drugs are available to treat PD, such as L-dopa, COMT inhibitor, MAO-B inhibitor, and dopamine agonists, but these drugs simply compensate for dopamine loss in PD and therefore cannot completely suppress its symptoms or progression. Drugs that are currently available for PD do not result in the much-expected permanent therapeutic benefits to patients, and as such scientists have now focused their attention towards natural compounds, which may have a more promising antiparkinsonian potential [18,19]. Several studies have documented that varieties of medicinal plants and natural bioactive compounds exhibit healing properties with negligible side effects via extraordinary antioxidant and anti-inflammatory action, which make them suitable candidates for antiparkinsonian activity [20,21].

Specifically, phytochemicals execute their effect against PD by several mechanisms, such as suppressing apoptosis (minimizing the level of caspase-3, -8, and -9, Bax/Bcl-2), synuclein deposition, reducing the loss of dopaminergic neurons, expression of proinflammatory cytokines (like interleukin-1β, nuclear factor-κB, prostaglandin E2, and interleukin-6), dopamine depletion, cellular inflammatory signaling, augmenting antioxidant status, and neurotrophic factors, respectively [20,22,23]. To date, different types of bioactive molecules have been extracted and recognized from plants including polyphenols, terpenes, and alkaloids. Surprisingly, there are no concise elaborated articles comprising updated reviews of published literature towards emerging trends in potent natural bioactive compounds for the management of PD. Therefore, we designed the current review to provides an illustration of the efficacious role of various medicinal plants and bioactive compounds (polyphenols, terpenes, and alkaloids) that can become potential therapeutics against PD with greater specificity, target approachability, bioavailability, and safety to the host. 

## 2. Pathophysiology of Parkinson’s Disease (PD)

**Figure 1:**
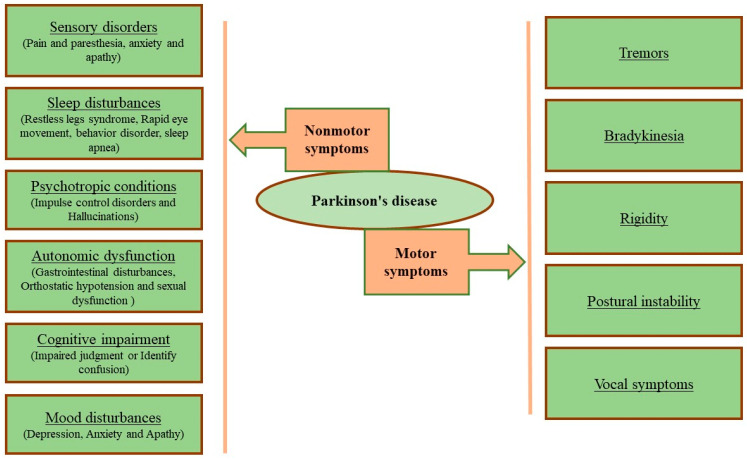
The diverse nature of various motor and non-motor symptoms affecting Parkinson’s disease (PD) patients (adapted from [24,27]).

**Figure 2:**
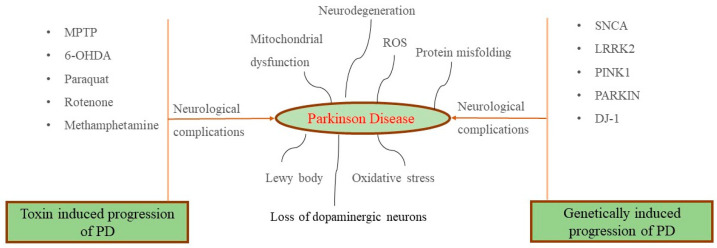
Toxicological model for pharmacological screening of investigated molecules against PD [31,32].

PD is defined as a neurodegenerative disorder showcasing different types of pathophysiological symptoms owing to A9 dopaminergic (DA) neuron loss in the SNpc selectively and formation of an intracellular aggregate called a Lewy body [12,24]. In pathological inclusions, α-synuclein (α-syn) shortened at the carboxy terminal congregates, thereby leading to its accumulation. Hemoglobin (Hb) expression is not only limited to erythrocytes but also evident in neurons. It is particularly concentrated in PD-susceptible mesencephalic dopaminergic neurons rather than resistant neurons. In physiological condition, the neuronal Hb has been found to be extensively restricted to cell bodies, including the nucleus, mitochondria, and the cytoplasm. However, it has been reported that when dopaminergic neurons were challenged with α-syn, Hb-α-syn complex formation was initiated in the mitochondria and cytoplasm, together with reduced transport of Hb from cytoplasm to the mitochondria, low levels of free mitochondrial Hb, and Hb aggregation in the nucleus, contributing to neuronal cell damage and pathological progression of PD. Research findings have shown that, due to the overexpression of Hb, cells’ vulnerability increases in an in vitro PD model and creates nucleolar and cytoplasmic aggregates in mice [12,25,26]. The dysfunctionality of the nerves becomes evident with a very broad range of non-motor symptoms, such as autonomic dysfunction, sleep disturbances, sensory abnormalities, and cognitive impairment together with neuropsychiatric symptoms. Figure 1 shows that PD is a multifaceted disorder influencing both motor as well as non-motor symptoms during the development of the disease [12,25,26,27,28,29]. In relation to the advent of various symptoms, PD can be categorized into three stages including preclinical, prodromal, and, clinical, respectively. In the preclinical stage, a fixed neurodegeneration is initiated in the SN, with no noticeable clinical symptoms. It is followed by a more than 10 years of a prodromal stage having unceasing neuronal loss but also showing some non-motor symptoms. Subsequently, a stage at which 40–60% of dopaminergic cells become non-functional followed by motor symptoms (tremors, rigidity, and bradykinesia) are characterized as the initial stage of PD to the patients. As evidence from various studies the genetic, environmental, and life-style components are some of the major influential factors for the pre-clinical stage. At stage 2, the promising biomarkers for early detection and diagnosis of PD include α-synuclein measurements in the cerebrospinal fluid, blood, and peripheral tissue and dopamine transporter scanning, respectively. Finally, during clinical stages, the evidence related to Lewy bodies’ development and multiple system atrophy are identified as a biomarker [30]. Further, several toxicological models, which have been established for pharmacological screening of several medicinal plants, bioactive compounds, and pharmaceutical drugs are herewith incorporated in Figure 2. 

## 3. Medicinal Plants, Herbal Formulations and Plant-Based Bioactives (Polyphenols, Terpenes, and Alkaloids) as a Potent Therapeutics for PD Management

Medicinal plants possessing high concentrations of additional nutritional elements provide both health advantages and increased nutritional value due to their potency to influence metabolic processes. In the recent decade, medicinal plants and bioactive constituents with diverse structures have shown to be promising resources for PD drug research. The efficacy of some notable medicinal plants and herbal formulations investigated in the last five years (2019–2023) with a mechanism of action for the management of PD has been incorporated in Table 1. The information on earlier published studies before 2019 has been already reviewed by many scholars [33,34,35,36,37,38,39]. Similarly, many polyphenols, terpenoids, and alkaloids manifest their possible beneficial properties in an in vitro and in vivo models of neurodegenerative disorders, specifically PD, as represented in Table 2.

### 3.1. Polyphenolic Compounds

Polyphenols are a class of significant natural bioactive molecule has been found to be broadly distributed in dietary vegetation and display potential neuroprotective properties against neuroinflammation and neuronal death as evidenced from both in vitro and in vivo experimentation. They have been further classified as flavonoids and non-flavonoids consisting of bioactive compounds including stilbenes, lignans, phenolic acids, curcuminoids, and coumarins.

Polyphenols are secondary metabolites synthesized in plants by the polyketide or shikimate pathway and commonly found in vegetables, fruits, nuts, and seeds. They possess multiple phenol units (C_6_H_5_OH) with hydroxyl groups (OH) linked to the aromatic benzene ring. Polyphenols have been found to exhibit potential therapeutic properties. A polyphenols-rich diet has demonstrated significant modulatory effects on the pathophysiological mechanisms of many underlying chronic diseases, especially in diabetes and cardiovascular and neurodegenerative diseases as evidenced from several experimental and clinical studies, indicating their significant prophylactic and therapeutic potential [82]. A growing body of evidence has demonstrated that the supplementation of polyphenolic compounds limits the risk for neurodegenerative disorders. Several of these compounds have been found to have cell-protective abilities against oxysterols (e.g., 7-ketocholesterol),mitigate mitochondrial dysfunction and cell injury. Polyphenols, namely resveratrol, quercetin, and apigenin, have shown potential scavenging activity against reactive oxygen species (ROS) induced by oxysterols and thereby counteracting ROS [83]. They possess influential antioxidant properties owing to their free radical scavenging potential and iron chelating action. Additionally, these have been further documented to display antiviral, antibacterial, anti-inflammatory, anticarcinogenic, and neuroprotective properties [84]. Recently, it has been established that plant polyphenols orchestrate neuroprotective abilities such as the capacity to combat misfolded protein gathering, the probability to endorse cognition, memory, ROS, neuroinflammation, neurotrophin secretion, and the capability to shield nerve cells after neurotoxins exposure [85]. These compounds possess at least one OH group existing over the aromatic side chain with its backbone having a simple moiety to a multifaceted polymer [86].

Structure activity relationship

Polyphenolic compounds contain an OH group at an ortho or para position acting as a hydrogen donor and reductant during redox reactions [87]. Antioxidant activity increases with the higher number of total OH groups present. Therefore, polyphenols counteract the oxidation of biomolecules by donating protons rapidly to radicals or by reacting with them to form products that block them from reacting with other biological molecules. Moreover, polyphenols also have the ability to interact with enzymes or receptors in signal transduction, thus modulating cellular oxidation and enhancing the antioxidant status [88]. Preclinical and clinical studies strongly support the protective action of polyphenols in neurodegenerative diseases owing to their high antioxidant activity orchestrated by the presence of OH groups in their structure [89].

In vitro and in vivo studies

Recent studies have demonstrated that dietary polyphenols reduce the breakdown of monoamine oxidase A (MAO-A)- and monoamine oxidase B (MAO-B)-dependent monoaminergic neurotransmitters, maintaining the levels of dopamine and serotonin in animal brain tissue [90]. More specifically, MAO-A is responsible for the metabolism of tyramine, norepinephrine (NE), serotonin (5-HT), and dopamine (DA). However, MAO-B mainly metabolizes DA and some less clinically relevant chemicals [91]. In this context, the supplementation of polyphenols to rats after post-fluid percussion injury demonstrated a promising neuroprotective action [92]. Further, research findings have also revealed that a polyphenol-rich diet normalizes brain-derived neurotrophic factor (BDNF) levels and synapsin 1-dependent synaptic plasticity. These studies support the role of polyphenols in the enhancement of memory, learning abilities, and hippocampal neurogenesis. Thus, polyphenols maintains normal brain health by directly influencing the central nervous system and the underlying machinery [93,94].

Polyphenols have been chiefly divided into two categories, namely flavonoids and non-flavonoids in accordance with current recognized classifications [95]. On the basis of the oxidation state and hydroxylation mode, the flavonoids have been further subdivided into flavanones, anthocyanins, flavanols, isoflavones, and flavones, whereas the non-flavonoids are further classified as stilbenes, phenolic acids, phenolic alcohols, lignans, coumarins, and curcuminoids [96,97,98].

#### 3.1.1. Flavonoids

**Figure 3:**
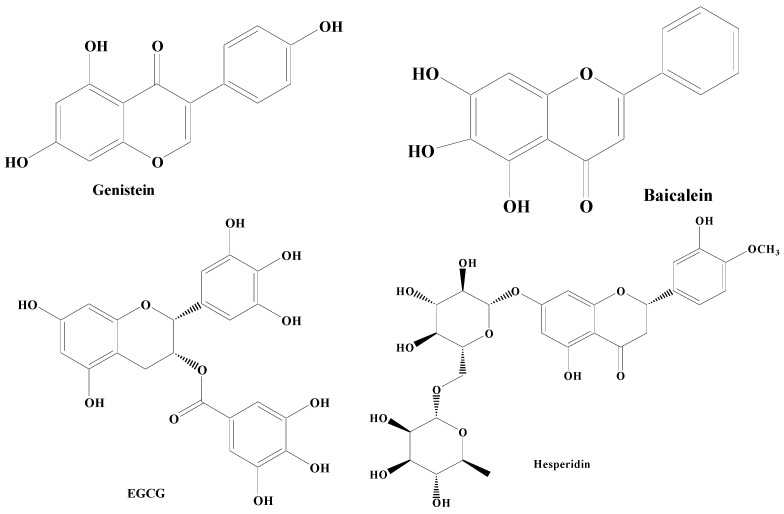
Different types of flavonoids.

Flavonoids have a common 1,2-diphenylpropane or 1,3-diphenylpropane (C6-C3-C6) basic structure [99]. Various biological properties of flavonoids include antithrombotic, anticancer, anti-inflammatory, antimicrobial, antiviral, and immunomodulation. Flavonoids have been reported in a wide variety of vegetables (tomatoes, onion, cabbage, cauliflower) and fruits (apple, grapes, berries, banana). Li et al. have revealed that these compounds are beneficial to skeletal muscles, liver, pancreas, adipocytes, and neuronal cells [100]. The results of the randomized clinical trials have demonstrated the enhancement of cognitive abilities resulting from the dietary intake of flavonoids-rich foods, irrespective of age and health status [21]. According to structure activity relationship studies, it has been documented that the double bond between the second and third carbon atoms, the 3′,4′-catechol, the ketone group, and the hydroxyl group at the third position present in the flavonoid backbone are responsible for the free radical scavenging and antioxidant properties of flavonoids. Because the double bond between C2–C3 is conjugated to the carbonyl group present in the C ring, unsaturated flavonoids have a higher capacity to scavenge free radicals in relation to saturated compounds like flavanones [101,102]. Mittal et al. mentioned that the extract of *Ginkgo biloba* rich in flavonoids orchestrates a protective impact on dopaminergic neurons in animal models of PD [21]. In vitro and in vivo findings suggest that flavonoids intake (with supplements or with normal diet) could be a promising intervention for the attenuation and/or prevention of the deterioration effects of cognitive decline associated with various neuronal disorders [22]. Flavonoids consists of potent bioactive compounds including genistein, baicalein, epigallocatechin-3-gallate (EGCG), and hesperidin (Figure 3).

Acacetin is a flavone found naturally in plants including *Linaria* spp., *Chrysanthemum morifolium*, *Calaminth* spp., *Carthamus tinctorius*, *Turnera diffusa* (known as damiana), and *Robiniapseudo acacia* (also called black locust) [103,104]. Findings of various studies have reported that neuroinflammation is not only involved in inflammatory diseases but also in neurodegenerative diseases, including PD. Neuroinflammation in PD is associated with microglial and T-lymphocyte activation with an upregulation of pro-inflammatory cytokines like prostaglandin E2 (PGE2), tumor necrosis factor-α (TNF-α), and nitric oxide (NO). Experiments conducted in rodent PD models have suggested that neuroinflammation is prominently implicated in neuronal cell death. Acacetin displays antiparkinsonian activities by diminishing the inflammatory factors associated with the inflammation. It also helps in tumbling dopamine-producing nerve cells, cyclooxygenase-2 (COX-2) glial stimulation, intensifying DA levels and inducible NO synthase (iNOS) [105,106].

Baicalein is one of the main flavonoids and has been reported to be found in roots of the Chinese medicinal herb *Scutellaria baicalensis* [107]. It has a wide array of biological functions, namely anti-inflammatory, antioxidant, antiviral, anticancer, and cardioprotection [108]. It also inhibits acetylcholinesterase and has neuroprotective ability. Baicalein exhibits antiparkinsonian activity by defending PD through caspase-mediated cell death inhibition and by increasing the feasibility of the SHSY5Y cell line. Specific proteins are also suppressed by this flavonoid, via controlling the neuronal cell damage and the ratio of Bcl-2-associated X protein linked with X protein/B-cell lymphoma (Bax/Bcl-2). It was further reported that baicalein potentially reduces the ROS generation, ATP deficit, apoptosis, and mitochondrial transmembrane breach in PC12 cells, when subjected to rotenone-induced neurotoxicity. Treatment with baicalein increases and maintains basal ganglia dopamine and 5-hydroxytryptamine levels. Additionally, it also decreases the oligomerization and aggregation of α-syn in SH-SY5Y and Hela cells [109,110,111].

Epigallocatechin-3-gallate (EGCG), an essential polyphenolic compound from green tea, has been found to function as an important therapeutic for the treatment of PD [112]. The antiparkinsonian outcome from EGCG is controlled through a rise in reactivators including coactivator-(PGC-1α), peroxisome proliferator-activated receptor, and SIRT1 protein expression. Hence, these are amongst the important metabolic supervisory transcriptase agents which are destined to have an input displaying the inflection in the cellular performance of cells in the anxiety state of PD [113,114].

Theaflavin (TF), is a major constituent of black tea, consisting of three vital compounds, namely TF-3,3′-digallate, TF-3′-gallate, and TF-3-gallate, responsible for lessening the adverse effect in SN TH-positive neurons and employing anti-apoptotic action via suppressing the activities of caspase-9, -8, and -3 in SN [115]. Various scientific studies have been carried out which demonstrated TF to possess excellent neuroprotective role against PD. TF has also been reported to eradicate toxic amyloid deposits. When compared to EGCG, TF3 was less susceptible to air oxidation and had an increased efficacy under oxidizing conditions. Interestingly, TF has been found equal in efficiency to EGCG at inhibiting β-amyloid and α-synuclein-induced neurotoxicity due to its potential antioxidant properties [116,117].

Fustin is a flavanonol extracted from *Rhus verniciflua* (heartwood). The flavanonol fustin orchestrates its neuroprotective ability by suppressing cell apoptosis, which is further facilitated by the drop in p38 phosphorylation, ROS generation, caspase-3 activation, and the Bax/Bcl-2 ratio [118]. 

Hesperidin, a flavanone glycoside, has also been found to be associated with multiple neuroprotective activities including the suppression of neuroinflammation, inhibition of oxidative damage, and anti-apoptosis [119]. It diminishes iron-induced death, mitochondrial dysfunction, OS, and reinstates levels of dopamine in the *Drosophila melanogaster* model of PD. Furthermore, hesperidin also prevents neuroinflammation which was attributed by the augmented synthesis of transforming growth factor-(TGF-) β and IL-10 in an MS mouse model. It possesses the ability to cross the blood–brain barrier (BBB) which can offer its application as a capable therapeutic towards the treatment and management of neurodegenerative diseases [120,121,122].

Anthocyanins are water-soluble flavonoids extensively found in numerous vegetables, as well as fruits such as purple grapes, blackcurrants, blueberries, cherries, and raspberries, respectively. Several studies conducted on cell lines, animal models, and humans have indicated that anthocyanins orchestrate anti-carcinogenic, anti-diabetic, cardiovascular disease prevention, and brain homeostasis [123]. Research findings have documented that anthocyanins exhibits neuroprotective potential through Aβ-inhibition, suppression of inflammatory responses, and reduction in oxidative damage [124,125].

Genistein, an isoflavone derived from *Glycine max*, has been explored for its implications in various diseases. This compound has attracted attention owing to its pharmacological roles, such as neuroprotection, cardioprotection, anti-cancer, antioxidant activity, anti-inflammatory effects, and obesity prevention. Recently, the synergistic effect of galantamine and genistein was evaluated to explore its neuroprotective ability against Aβ1–42-triggered toxicity in AD. The results of the study demonstrated decreased genotoxicity and cell death by influencing the RAGE/LRP-1 pathway in Wistar rats. Genistein has been extensively studied for its neuroprotective potential in numerous neurodegenerative disorders including PD [126,127,128].

#### 3.1.2. Non-Flavonoids

Non-flavonoids are also polyphenolic compounds exhibiting various types of neuroprotective effects in PD by different mechanisms of action. This group of compounds comprises phenolic acids, phenolic alcohols, stilbenes, and curcumin.

##### Phenolic Acids

**Figure 4:**
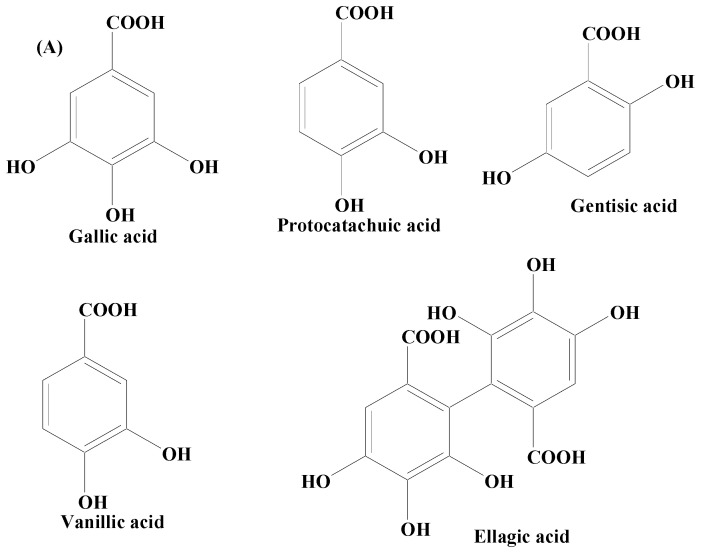

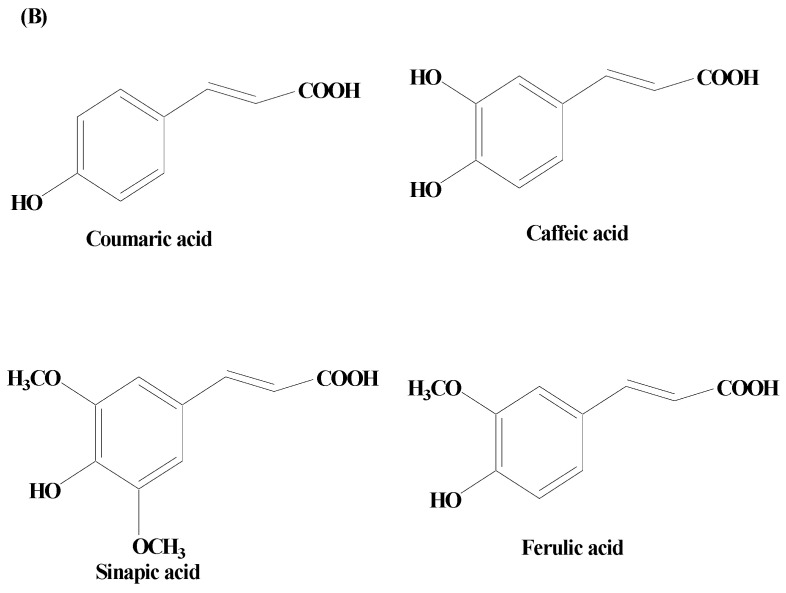
(**A**) Phenolic acids—hydroxycinnamic acid. (**B**) Phenolic acids—hydroxybenzoic acid.

Phenolic acids are secondary metabolites produced by plants. They exist as acidic complexes in the form of hydroxycinnamic and hydroxybenzoic acids (Figure 4A,B) [129]. They possess important biological and pharmacological properties including anti-inflammatory, anticarcinogenic, anticancer, antioxidant, and antimutagenic. Due to the presence of the phenol group and resonance-stabilized conformation, phenolic acids stand out among other chemicals of natural origin for their potent antioxidant and anti-inflammatory activity, which is achieved by a radical scavenging mechanism. Studies carried out using PC12 cells have demonstrated that phenolic acids mitigate 1-methyl-4-phenylpyridinium (MPP+)-induced cell death by boosting the neurite network and triggering the production of proteins essential for synaptogenesis (synaptophysin and synapsin I) and axonal growth (GAP-43). Findings of studies have also demonstrated that phenolic acid reduces the sickness behavior and neuroinflammation induced by lipopolysaccharide (LPS) in mice. TNF-α, a measure of inflammation, has been found to be decreased in the serum in a dose-dependent way, when phenolic acids were administered orally (30 mg/kg) one hour before LPS exposure (1.5 mg/kg) [130]. Phenolic acids are composed of a wide array of bioactive compounds including gallic acid, coumaric acid, ellagic acid (EA), salvianic acid, and rosmarinic acid.

Gallic acid (GA) is a phenolic acid widely distributed in grapes, berries, nuts, honey, tea, and vegetables, either in the bound or free form as a derivative. It has been utilized in a variety of healthcare conditions and has been shown to be effective in preventing stroke, cardiovascular problems, Parkinson’s disease, and Alzheimer’s disease. GA’s capacity to cross the BBB, scavenge abnormal levels of ROS and RNS, and bind transition metal ions are the underlying machinery for orchestrating its neuroprotective actions. GA has been demonstrated to end the vicious cycle of OS and tissue injury as a result of its scavenging capacity and the activation of important antioxidant enzymes in the brain. It was further shown that GA reduces neurobehavioral activities by lowering interleukin-1, nitric oxide, myeloperoxidase activity, and TNF-α, and increasing glutathione levels, antioxidant activities, lowering OS, and caspase-3 levels. It also checks apoptosis by bringing down the levels of caspase 3. GA supplementation further enhances the neuromotor abilities that deteriorate during psychosis. It has been found to be promising in reducing lipid peroxidation, controlling dopamine levels, and inflammatory signals [131,132,133].

A polyphenolic compound, para-coumaric acid (p-CA), is a plant-derived secondary metabolite. p-CA is a dietary polyphenol distributed in several natural food sources, such as tomato, carrot, green pepper, and strawberry, functioning majorly as an antioxidant [134,135]. However, a growing body of evidence has reported its beneficial effects including anti-inflammation, antihyperlipidemia, antihyperglycemia, antineurodegeneration, anticancer, anticardiac infarction, and antimicrobial. Research findings have demonstrated the potent neuroprotective potential of p-CA. In vivo findings have shown that p-CA supplementation diminishes the degeneration of axon in sciatic nerves of rat and OS, followed by ischemia or reperfusion [136]. p-CA has also exhibited neuroprotective potential in models of global and local cerebral ischemia by preventing apoptosis and ROS generation [137].

Ellagic acid (EA) is a phenolic compound extensively distributed in dicotyledonous plants possessing robust antioxidant and anti-inflammatory properties. Research also revealed that it improves neural feasibility, lessens neuronic faults, and decreases injury associated with neurodegenerative diseases [138]. EA has also been found to mitigate oxidative damage by controlling the pathways like Nrf2 and NF-κB and also by refining the antioxidants, as well as antioxidant enzymes’ action. Further, findings have revealed that EA could promisingly reduce malondialdehyde heights and amplify proceedings of total GSH, catalase, and superoxide dismutase (SOD) in a PD animal model [139,140].

Salvianolic acid B is obtained from *Salvia miltiorrhiza* plant. Research findings have suggested that this compound is potentially capable in amending the rate of cell death. Further, it orchestrates its bioactive role through various ways including maintenance of ROS levels, releasing alteration of cells nuclear morphology protecting matrix metalloproteases, tempering cell’s apoptotic and antiapoptotic mediators, and dropping Bax/Bcl-2 ratio, thereby lowering caspase-3 enzyme activity [141,142,143].

Syringic acid is a naturally occurring derivative of benzoic acid distributed widely in edible plants and fruits. It exhibits antiparkinsonian potential by reducing lipid peroxidation, refining the GSH level, and conquering the pro-inflammatory expression of cytokines including COX-2 enzyme, interleukin (IL)-β1, and TNF-α. Motor dysfunction is prevented by means of striatal DA damage and also their metabolites in MPTP-induced experimental model and consequently, the expression level of VMAT-2 and TH inside the SN is amended [144,145,146].

Rosmarinic acid, an important phenolic compound derived from cinnamate is reported in several naturally occurring plants, namely *Melissa officinalis*, *Ocimum basilicum*, *Salvia officinalis*, *Origanum majorana*, and *Rosmarinus officinalis*. It has displayed an outstanding antiparkinsonian action by improving the viability of cells, shielding matrix metalloproteases through intracellular ROS production hindrance, elevating DA levels, and controlling the ratio of Bcl-2/Bax. Rosmarinic acid has also been demonstrated to positively influence cell nuclear condensation, the mitochondrial respiratory chain, and several cell morphological fluctuations. In addition, it also helps in the deactivation of caspase-3 which in turn helps in re-establishing the activity of complex I in the mitochondrial electron transport chain [147,148,149].

##### Phenolic Alcohols

Phenolic alcohols reported in various plants are also polyphenolic compounds containing an OH group linked to an aromatic hydrocarbon. Plants synthesize this class of compounds in order to combat environmental stresses including pathogens or insects that attack plants [150,151]. Phenolic alcohols orchestrate antioxidant and anti-inflammatory activities by rescuing nerve terminals in the striatum and dopaminergic neurons in the SNpc area and restoring SOD, CAT, and glutamate levels, preventing lipid oxidation, reducing the level of ionized calcium binding adaptor molecule (Iba-1), GFAP hyperactivity, pro-inflammatory cytokines, iNOS, and COX-2 activities, respectively. They contain an OH moiety linked to an aromatic hydrocarbon which helps to scavenge free radicals and protect the neuronal damage. In vitro and in vivo studies have shown that phenolic alcohols have significant levels of anti-inflammatory and antioxidant properties due to which they play a very important role in managing PD symptoms [152]. The major phenolic alcohols found predominantly in plants are 6-Shogaol and sesamol.

6-Shogaol (6S) is a pungent ingredient extracted from ginger. It exhibits a wide range of pharmacological properties including neuroprotective potential by overpowering neuroinflammatory factors including TNF-α, NO, COX-2, and iNOS. In addition to these, it also acts through the activation of microglia in the SNpc. Several investigations have documented its neuropharmacological effects for neurodegenerative disorders. In an AD transgenic mice model, 6S prevented aberrant buildup of the Aβ-peptide in the hippocampus and cortical areas and improved memory impairment. Additionally, it has been documented to reduce memory loss, neuronal damage, and neuroinflammatory effects in mice. In studies on 1-methyl-4-phenyl 1,2,3,6-tetrahydropyridine (MPTP)-induced PD models, the anti-PD effects of 6S have been investigated. These studies revealed that 6S remarkably prevents dopaminergic neuronal damage, MPTP-induced motor impairment, and striatal dopamine depletion. In MPTP-induced PD mice, 6S prevents gliosis, dopaminergic neuronal degeneration, and motor impairment. Further, it prevents nuclear factor B’s increased nuclear translocation, as well as apoptotic cell death [153,154,155,156].

Sesamol, a lignin obtained from the shrub *Sesamum indicum*, holds a wide range of defined neuroprotective potential, and remains utilized as therapeutics against PD. Owing to high metabolic activity, brain requires a lot of energy inputs for physiological functions. The brain also features a high membrane surface to cytoplasm ratio, a low repair capability, a non-replicating nature of neurons, and a comparatively low antioxidant machinery. Due to an imbalance between pro-oxidant and antioxidant agents in the brain, increased free radicals, which are mostly created through oxidative phosphorylation, play a significant role in neurological illnesses. This supports the need to target antioxidant systems in order to combat OS and the resulting brain disorders. By encouraging antioxidative defense systems for neutralizing free radicals and by limiting transcription, the antioxidant system is crucial for saving neuronal cells from OS and maintaining the proper redox balance in the brain tissue. Sesamol has been found to boost the action of various antioxidant enzymes, namely glutathione reductase, CAT, SOD, GPx, and also non-enzymatic antioxidants (vitamin E, GSH, and vitamin C), thereby reducing the levels of the lipid peroxidation and nitrites [157,158,159,160].

##### Stilbenes

Stilbenes are polyphenolic compounds found in various plant species. They have been shown to have anti-inflammatory properties, estrogen receptor agonist qualities, and effects on cell proliferation, cell signaling pathways, and apoptosis. Stilbenes also exhibit antifungal, antiviral, and antibacterial properties. They possess numerous beneficial attributes for the inhibition of abundant pathophysiological issues, namely age-linked ailments (example: type 2 diabetes mellitus, and obesity), OS, and neurodegenerative disorders including PD [161,162]. In addition, an oligostilbene compound, Amurensin G, found in *Vitis amurensis* (a type of wild grape) root was revealed to maintain the survivability of SH-SY5Y cells by downregulating α-syn and ubiquitinated proteins [163,164].

**Figure 5:**
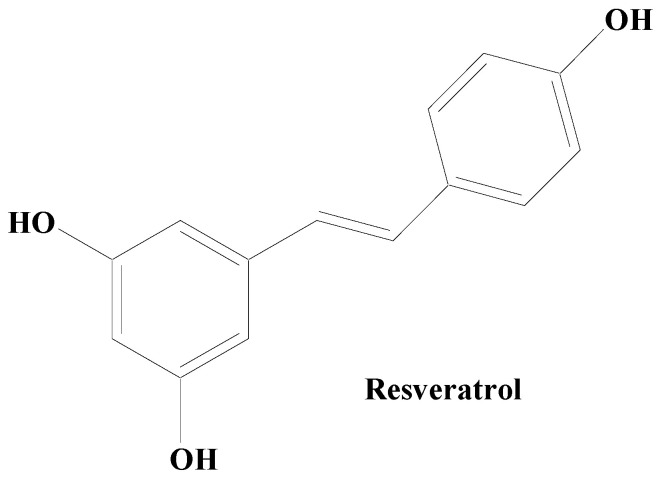
Natural stilbene—resveratrol.

A stilbene compound, resveratrol, occurring naturally in several plants including *Polygonum cuspidatum* has demonstrated protective potential in the animal and cellular models of PD (Figure 5). Heme oxygenase-1 (HOX-1) is a 32 kDa stress response protein implicated in the prevention of PD. HOX-1 functions in response to stress and eventually becomes downregulated. Resveratrol exhibits neuroprotection against paraquat-induced PC12 cells via HOX upregulation. Supplementation of resveratrol has been found to upregulate the SOD enzyme activity [165]. The in vitro findings on antiparkinsonian activity of this compound have revealed the capability of resveratrol to downregulate the level of caspase-3 enzyme activity and the lactate dehydrogenase (LDH) leakage. On a similar line, its hydroxylated derivative oxy-resveratrol also has reported neuroprotective potential through the decrease in ROS generation inside the cell, reduction in phospho-JNK-1 and 2, and cytosolic SIRT1. Due to its hydroxyl group and competing with coenzyme Q, resveratrol decreases the activity of complex III, hence lowering the formation of ROS. Resveratrol has also been demonstrated to protect oxygen glucose deprivation and reperfusion (OGD/R), at least in part, according to a study on the PC12 cell line. It exerts neuroprotective effects by reducing OGD/R-induced OS and maintaining mitochondrial function through PINK1/Parkin-mediated mitophagy [103]. Studies carried out using two-month-old male rats with middle cerebral artery occlusion (MCAO) treated with rehabilitation training and resveratrol indicated that resveratrol enhanced neurological and motor function in MCAO rats via activating the brain-derived neurotrophic factor/tyrosine kinase receptor B (BDNF/TrkB) signaling pathways and SIRT1 signaling network. In both in vivo and in vitro experimental models of neurodegeneration, studies have shown that resveratrol moderates mitochondrial activity, maintains redox homeostasis, and cellular dynamics [166].

##### Curcumin

**Figure 6:**
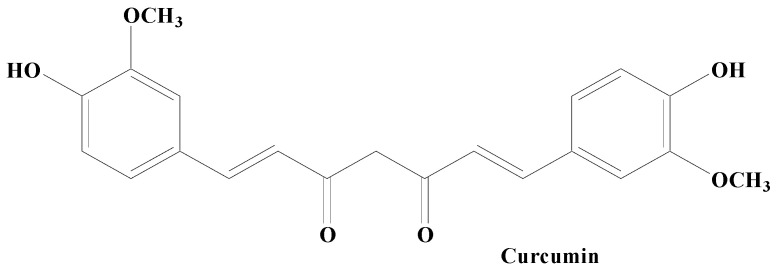
Curcumin.

Curcumin, also known as diferuloylmethane is a polyphenolic compound obtained from the plant rhizome *Curcuma longa*. Curcumin exhibits, a broad array of pharmacological properties owing to its hydroxy (antioxidant activity) and methoxy (antitumor and anti-inflammatory activities) groups (Figure 6). It has been established to possess various health benefits. Curcumin’s therapeutic and prophylactic efficacy has been demonstrated in many neurodegenerative, oncological, inflammatory, and autoimmune diseases. Studies carried out on an antiparkinsonian rat model revealed that curcumin treatment potentially controls the PD complications via the dopaminergic neuronal damage and depletion of DA. Moreover, iron chelating activity in addition to condensed iron-positive cells in SN has also been mitigated by curcumin. Research findings have shown that curcumin has the ability to downregulate the level of the caspase-3 enzyme, besides amplifying the LRRK2 mRNA and protein expression in vitro. Accumulated evidence showed that curcumin exhibits various neuroprotective properties, including chelating metal ions’ antioxidation, inhibition of the aggregation of misfolded proteins, and attenuating neuroinflammation [167,168]. According to Jin et al., curcumin was found to alleviate PD by energizing the BDNF/PI3k/Akt transduction pathway [169]. Additionally, a nanoformulation of this medication plus levodopa was recently suggested for the treatment of PD [170]. In a PD model, curcumin also offers protective effects on the cerebellum [171]. Earlier biochemical findings established that curcumin proficiently blocked aggregation of α-syn in vitro. Various improved equivalents of curcumin having enhanced constancy have also been confirmed to be effective in preventing depolymerizing α-syn fibrils and α-syn amyloid aggregation. In vivo studies have demonstrated that curcumin has no effect on how α-syn condensates develop or their initial shape. It does however effectively prevent α-syn from amyloid genesis by reducing its fluidity within the condensates. Additionally, it prevents α-syn E46K and H50Q mutants that are linked to PD illness from aggregating amyloid under phase separation. Curcumin can also weaken α-syn amyloid aggregates that have already developed in the condensates [167,168].

### 3.2. Terpenes

**Figure 7:**
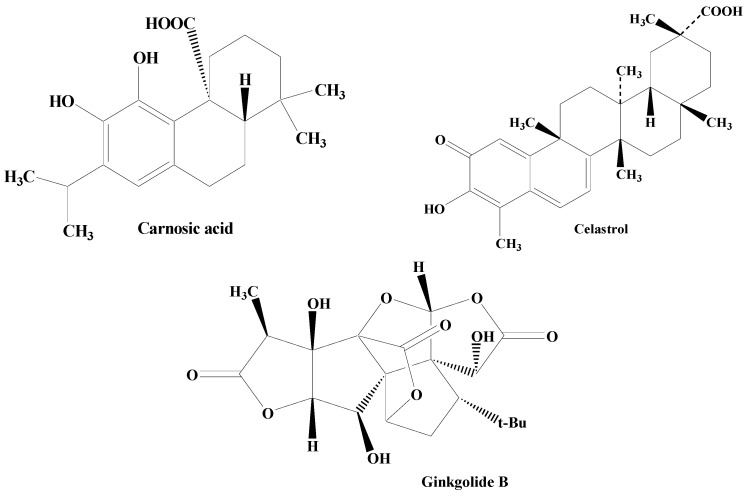
Structures of various terpenes.

Terpenes are one among the most widely distributed compounds in the plant kingdom. They possess the utmost molecular dissimilarity amongst the secondary metabolites. Terpenes are mainly obtained from coniferous plants, namely juniperus, abies, pinus, and picea. They are mainly hydrocarbons constituting the main bioactive components of natural products including essential oil, wax, rubber, and resin. Terpenoids have biological and pharmacological potential including anticancer, antiviral, anti-inflammatory, antifungal, antihyperglycemic, antiparasitic, and antimicrobial [172,173,174,175]. Some of the important terpenes includes carnosic acid, ginkgolide B, and celastrol (Figure 7).

Carnosic acid, reported in the herb rosemary, is a phenolic diterpene. It amplifies neural cell capability by enhancing the antioxidant presentation in cellular models of cell apoptosis, and also through interacting with the γ-glutamyl cysteine ligase catalytic subunit, SOD, and GSR stimulation of nuclear factor-E2-linked factor 2 (Nrf2) pathways, brain-derived neurotrophic factor (BDNF) release, and γ-glutamyl cysteine ligase modifier subunit [176,177,178].

One of the important diterpenes, Ginkgolide B, extracted from *Ginkgo biloba* shields against neuronal damage by lowering the calcium concentration within the cell, declining the action of caspase-3 enzyme and cell death. The calcium-binding protein calbindin D28K encourages neuronal process extension in dopaminergic neurons, thereby possessing the potential to defend dopaminergic neurons against uncontrolled PD. The terpene ginkgolide B holds an outstanding capability of restoring the protein calbindin D28K mRNA, as evidenced by in vitro studies [179,180,181,182,183,184].

A triterpene celastrol found in *Tripterygium wilfordii* lessens the loss of dopaminergic neurons, thereby minimizing the DA and DOPAC level exhaustion indicating its antiparkinsonian potential. It is associated with the synthesis of various noteworthy intermediates in the inflammation like NF-κβ and TNF-α. Celastrol also orchestrates neuroprotective activity through the attenuation of the loss in the SNpc and dropping reduction in its levels in the striatum. In addition to this, it has also been found to augment the expression of HSP70 in the SNpc. Celastrol is reported to be involved in the nuclear translocation of cytoplasmic HSP70 facilitating HSP70 expression. After inducing expression of HSP70, inflammation is documented to be reduced through the prevention of TNF-α and NF-κB stimulation [185,186,187,188].

Structure Activity Relationship

Terpenes are a subclass of hydrocarbons that make up the majority of natural products like rubber, resin, wax, and essential oils. Structurally, terpenes are made up of isoprene units. The fundamental structure of terpenes, isoprene (2-methyl-1,3 butadiene), is made up of short carbon units with two double bonds and five carbon atoms. Terpenes have been classified based on the number of isoprene units. These units arrange themselves in the form of head–tail to form compounds with straight chains or rings. Due to the presence of isoprene in their structure, terpenes have been demonstrated to have antiparkinsonian, antimutagenic, antioxidant, and anticarcinogenic potential. As a result, they are frequently utilized in aromatherapy and phytotherapy. Terpenes have been employed in a variety of industries including food, medicine, cosmetics, pharmacy, and cleaning because of their multifaceted properties. Additionally, in recent years, it has been advised to use several terpenes derived from plants as supplements to enhance overall health [172,189,190].

In vitro and in vivo studies

In vitro investigation has showed that the terpene ginkgolide B has a remarkable capacity for repairing the protein calbindin D28K mRNA [170]. Investigations carried out in PC-12 and SH-SY-5Y cell lines demonstrated that ginkgolide B can promote antioxidant mechanisms via the Akt/Nrf2/ARE pathways. In vitro and in vivo studies have demonstrated that terpenes prevent demyelination and work with astrocytes to orchestrate a neuroprotective effect [191,192,193,194].

### 3.3. Alkaloids

**Figure 8:**
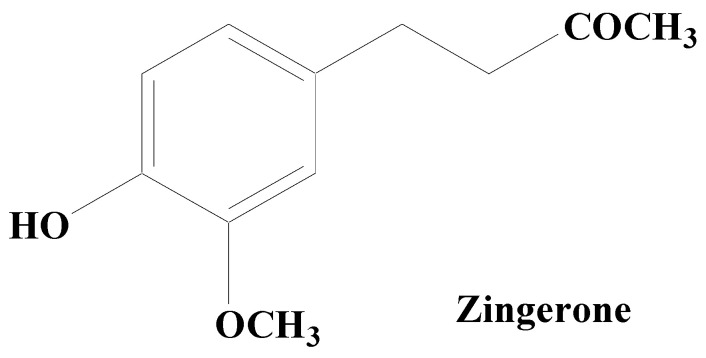
Zingerone.

Alkaloids are secondary metabolites, consisting of nitrogen, which at the beginning have been considered as the principal group of bioactive natural compounds isolated from plants (Figure 8). Additionally, alkaloids exhibit much structural diversity including in the chemical skeleton such as tetra-hydro-isoquinoline, indole, pyrrolizidine, tropane, piperidine, quinolizidine, indolizidine, pyridine, pyridinone, quinoline, quinazoline, xanthine, steroid, terpenoid, chromone, and flavoalkaloids, respectively [195]. A wide variety of biological roles such as antidepressant, emetic, diuretic, antimicrobial, antiviral, antihypertensive, anti-inflammatory, antitumor, anticholinergic, myorelaxant, hypoalgesia, and sympathomimetic have been displayed by alkaloids [196]. Various studies have shown that numerous alkaloid components possess a promising relaxing ability for a diverse neuron-related disorders including PD [197]. Thus, natural product-based alkaloids having polypharmacology variation characteristics are very beneficial in the progress of drug development in managing PD [198,199,200]. Zingerone and acetylcorynoline are among the important alkaloids possessing diverse pharmacological functions.

Zingerone, an alkaloid component found in the rhizome of ginger has been established to possess an outstanding antiparkinsonian potential. By lowering the expression of glial fibrillary acidic protein and IL-1ß in the hippocampus, it inhibits the overactivation of astrocytes and attenuates LPS-induced neuronal cell death. Low antioxidant levels lead to the creation of free radicals (ROS/RNS) and the consequent inflammation is considered to be the major cause for neurodegeneration in PD. Zingerone potentially inhibits the inflammatory cascade components including TNF-α, NO, COX-2, and iNOS that may contribute to lowering memory impairment in animal models of dementia by limiting glial cell activation. It modulates the depletion of dopamine and their metabolic products, namely homovanillic acid and DOPAC. In addition, it also boosts the antioxidative defense like superoxide scavenging and hydroxyl action, with the suppression of OS. Zingerone pretreatment has been found to upregulate the dopamine levels in the nigrostriatal region, which clearly suggests its protective role in the management of PD and its associated symptoms [201,202,203,204,205].

Acetylcorynoline, an alkaloid obtained from *Corydalis bungeana*, has also been revealed several neuroprotective abilities including inhibition of dopaminergic neuron loss, augmented levels of α-syn, and exhaustion of DA level. Programmed cell death has been documented to be suppressed by dropping the abnormal-1 (egl-1) expression levels, an apoptosis regulator, exhibiting its antiparkinsonian potential. It is well reported that acetylcorynoline is able to prevent pathogenesis in PD via increasing protein breakdown by proteasomes. Acetylcorynoline has been found to facilitate the increased expression of rpn-5, a proteasome-governing subunit, suggesting its antiparkinsonian activity [206].

Structure Activity relationship

The alkaloids’ nucleus contains a benzene ring linked to several (three to four) ether bonds having the capacity to generate hydroxyl groups. However, as there are not many hydroxyl groups present on these compounds, they have a low degree of polarity, which makes it easier for them to cross the BBB. Multiple ether bonds are transformed into hydroxyl groups attached to the benzene ring, which makes these aromatic hydroxyl groups powerful antioxidant structures for crossing the BBB. Based on the structural perspectives, alkaloids may act primarily as antioxidants, with anti-inflammatory, autophagy modulation, and suppression of calcium overload effects [207,208,209,210].

In vitro and in vivo studies

Several studies have demonstrated a neuroprotective effect of alkaloids against H_2_O_2_-induced oxidative damage in SH-SY5Y cell lines and AChE inhibitory activity [211,212]. Studies conducted in vivo subjected to aluminum-induced neuroinflammation and excitotoxicity have revealed that alkaloid therapy was successful in controlling glutamate and acetylcholinesterase levels, which were otherwise raised by aluminum. The findings of this study also been demonstrated to reduce the excitotoxic harm induced by aluminum, as well as the level of expression of the inflammatory markers IL-6 and TNF-α. The increased expression of inflammatory markers in the groups that received alkaloid treatment is suggestive of the neuroprotective ability of alkaloids in the recovery of neuroplasticity. The neuroprotective ability of alkaloid is further supported by histopathology, wherein the therapy greatly reduced neuronal loss and degeneration while restoring healthy and viable neurons. The study’s findings support the hypothesis that alkaloid has neuroprotective properties against neuroinflammation and excitotoxicity brought on by aluminum [213,214,215].

## 4. Conclusions

PD stands second among the long-lasting neurological diseases which disturb both cognitive performance and motor skills. Different types of healing methods have been actively involved in the managing PD to date, but they only provide symptomatic relief. Therefore, exploration for achieving the innovative natural moieties from plant sources can be very helpful in lessening the psychological antagonistic properties, and can also recover efficiency through healing aids, common in the patients with PD: this is the emphasis of the current review. The present assessment demands that bioactive natural compounds of plants origin be taken into consideration, which can play a vital role in PD treatment and management.

## Figures and Tables

**Table 1:** Notable medicinal plants or herbal formulation investigated for management of PD in the last five years (2019–2023).

Name of Extract or Formulation	Dose	Study Model	Mechanism of Action
*Acanthopanax senticosus* extract	4.5 g/kg	MPTP-induced mice	Regulated multiple targets to improve motor deficits [40].
*Antrodia comphorata*	10–50 mmol/L (in vitro); 10, 50, and 100 mg/kg (in vivo)	6-OHDA induced MES23.5 cells and C57BL/6 mice	Downregulate NLRP3, ASC, IL-1β, caspase-1, and ROS and upregulate dopaminergic neuron protection [41,42].
*Asarum sieboldii*	5 μM	Olfactory cell line (hONS)	Induced significant perturbation on biological organelles [43].
*Cervus nippon* (Velvet antler from sika deer)	20–40 μg/mL and 30 mg/kg	In vitro (BV2 cells), *Caenorhabditis elegans*, and MPTP-treated mice	Decreased aggregation of α-synuclein and protect from oxidative stress-induced DAergic neuron degeneration [44].
*Codium tomentosum* enriched fractions	100 µg/mL	6-OHDA-induced SH-SY5Y human cells	Mitigation of ROS generation, mitochondrial dysfunctions, and DNA damage followed by reduction in Caspase-3 activity [45].
*Crossyne flava*	2.5, 5, and 10 µg/mL	MPP+-induced SH-SY5Y cells.	Inhibited ROS and ATP depletion followed by induction of apoptosis [46].
*Ganoderma lucidum* extract	800 μg/mL and 400 mg/kg	Neuro-2a cells and mouse model	Regulating autophagy, mitochondrial function, and apoptosis [47].
*Geranium robertianum* aqueous extract	0–200 µg/mL	MPP+-induced SH-SY5Y	Antioxidant and apoptosis inhibitory properties [48].
Hidrox^®^ with Hydroxytyrosol	10 mg/kg, i.p.	Rotenone induced mice	Improves neuroinflammation, oxidative stress, and apoptosis [49].
Liuwei Dihuang Pills (enriched with quercetin, stigmasterol, kaempferol, and β-sitosterol)	Not available	Network pharmacology (in silico)	Regulates AKT1, VEGFA, and IL6, G protein-coupled amine receptor activity, ROS, membrane raft, MAPK signaling pathway, and cellular senescence [50].
*Myrica esculenta* leaves methanol extract	50, 100, and 200 mg/kg, orally for one week	Haloperidol-induced rats	Escalation of cellular antioxidants [51].
Polyscias fruticosa leaves extract	1, 2, 4, 8, and 16 mg/mL	Drosophila melanogaster model (dUCH knockdown)	Ameliorate dopaminergic neuron degeneration [52].
*Sphaerocoryne affinis* fruit water extract	3, 6, 12, and 18 mg/mL.	DPPH and fly model	Ameliorate the locomotor disabilities and degeneration of dopaminergic neurons [53].

**Table 2:** Plant-based bioactives (polyphenols, terpenes, and alkaloids) in the management of PD.

Compounds	Botanical Sources	Dose	Study Model	Mechanism of Action
**Polyphenols**				
Quercetin	Onions, apples, tea, brassica vegetables, and nuts	30 mg/kg for 30 days	Acrolein (3 mg/kg for 30 days) induced rats	Protects cerebellum tissues from neurotoxicity and oxidative stress [54].
Apigenin	Grape fruit, parsley, celery, and oranges	50 mg/kg apigenin, 5 days	MPTP (25 mg/kg for 5 days) induced mouse	Reverses the expressions and concentrations of TNF-α, IL-1β, IL-6, IL-10, and TGF-β [55].
Chlorogenic acid	Coffee (*Coffea arabica*)	1, 5, 10, 20, 40, and 100 µg/mL (in vitro); 50 mg/kg, orally for 13 weeks (in vivo).	In silico, in vitro (GLUTag cell line), and in vivo (rotenone-induced PD mice).	Acts as GLP-1 secretagogue [56].
	Coffee, honeysuckle, and Eucommia	75, 150, and 300 μM	MPTP zebrafish (6-OHDA-treated SHSY5Y cells).	Boosting the autophagy in neuronal cells [57].
Hydroxytyrosol	Extra virgin olive oil	250 µg/mL	*C. elegans* models	Improvements in locomotive behavior and the attenuation of autofluorescence [58].
	Virgin olive oil	1, 10, 25, and 50 μM	Murine microglial BV2 cell line	Microglial activation, expression of NADPH oxidase, MAPKs, and production of ROS [59].
	Extra virgin olive oil	0.1–200 µM (in vitro) and 50 mg/kg (in vivo).	In silico, in vitro (platelet MAO-B activity) and MPTP-induced mouse model	MAO-B inhibition (IC_50_: 7.78 μM), improved DA and motor impairments [60].
	Methanol extract of *Buddleja cordata* and extra virgin olive oil	1.5 mg/kg	MPP+ induced rats	Inhibitory effect on the MAO isoforms (MAO A and MAO B) [61].
	Micellar nanocarriers	10–200 μM	In vitro (hCMEC/D3-SH-SY5Y) cells (rotenone)	Improves oxidative stress by 12% and 9%, respectively, compared to the corresponding free drug [62].
Schisandrin B	*Schisandra chinensis*	100 μM	6-OHDA-induced SH-SY5Y cells and mice	Inhibitis the negative modulation of miR-34a on Nrf2 pathway [63].
Ginkgolic acid	*Ginkgo biloba* leaves	10, 40, and 80 μM	KCl-induced SH-SY5Y cells	Promotes autophagy-dependent clearance of α-syn aggregates [64].
Pinocembrin-7-methylether	Pigeon pea, thai ginger, honey, and propolis	Up to 200 μM	6-OHDA-induced SH-SY5Y cells and zebrafsh	Activation of Nrf2/ARE/HO-1 signaling cascades [65].
Chrysin	Passion flowers (*Oroxylum indicum*, *Passiflora incarnata* and *Passiflora caerulea*), *Scutellaria baicalensis*, mushrooms, bee propolis, and honey	Up to 500 mg/kg	In various in vitro and in vivo models	Increasing the expression of Nrf2, activates MEF2D, suppresses the MPP-induced upregulation of c-caspase and Bax, as well as the downregulation of anti-apoptotic protein Bcl 2. Additionally, enhances the production of neurotrophic factors and increase dopamine levels in the striatum via MAO-B [66].
Vanillin	Natural vanilla	100, 200, 300, 400, and 500 nM (in vitro) and 5, 10, or 20 mg/kg (in vivo)	LPS-induced murine microglial BV-2 cells and rats	Reduces over expression of iNOS, COX-2, IL-1β, and IL-6 through regulating ERK1/2, p38 and NF-κB signaling [67].
Ferulic acid	*Rhizoma Ligustici wallichii*, *Angelica sinensis*, and *Asafoetida giantfennel*	50 μM	6-OHDA-induced *C. elegans* models	Autophagy induction [68].
Heptamethoxyflavone	Orange and grapefruit	3–10 µM (in vitro) and 1.2–100 mg/kg (in vivo)	In various in vitro and in vivo models	Regulates IL-1β expression and suppresses MK-801-induced locomotive hyperactivity [69].
Kaempferitrin	*Cinnamomum osmophloeum*	25 µM (in vitro) and 2–5 mg/kg (in vivo)	In various in vitro and in vivo models	Prevents H_2_O_2_-induced oxidative stress [69].
Vitexin	Hawthorn, pearl millet, mung bean, pigeon pea, mosses and tartary buckwheat sprouts	10–50 µM (in vitro) and 1–100 mg/kg (in vivo)	In various in vitro and in vivo models	Regulates PI3/AKT, mTOR pathway; enhanced effect of TPv1 and NR2B pathway, suppresses CDPK II, prevents lipid peroxidation by TBHP; and inhibits effect of CYP2C11 and CYP3A1 [69].
Amentoflavone	*Cnestis ferruginea*, Hypericum perforatum, *Viburnum* and *Ginkgo* species.	0.1–60 µM (in vitro) and 0.1–100 µg/mL (in vivo)	In various in vitro and in vivo models	Inhibits COX and phospholipase A2, activate p38-AKT signaling pathway and inhibits production of prostaglandins E2 [69].
Mangiferin	*Swertia minor* and *Mangifera pajang*	Up to 100 mg/kg	In various in vitro and in vivo models	Counteracts the neurotoxic effect of MPTP, rotenone, and 6-OHDA, etc. [70].
Myricetin	*Myrica nagi*	Up to 100 mg/kg	In various in vitro and in vivo models	Protective effect against amyloid-beta, MPTP, rotenone, and 6-OHDA, etc. [71].
**Terpenes**				
Asiatic acid	*Centella asiatica*	10–100 nM	LPS-induced BV2 microglia cells and MPP+-induced SH-SY5Y cells,	Protects dopaminergic neurons from neuroinflammation by suppressing NLRP3 inflammasome activation in microglia cells as well as protecting dopaminergic neurons directly [72].
Paeoniflorin	Herbaceous peony	30 mg/kg	Network pharmacology and MPTP-induced mice	Inhibits apoptosis in hippocampal neurons of the CA1 and CA3, and upregulates PSD-95 as well as SYN protein levels. Similar protective effects were observed upon JNK/p53 pathway inhibition using SP600125 [73].
Madecassoside	*Centella asiatica*	15, 30, 60 mg/kg	MPTP-induced rats	Reversing the depletion of DA, antioxidant activity, increasing ratio of Bcl-2/Bax, increasing protein expression of BDNF [74].
Loganin	*Cornus officinalis* fruits	50 mg/kg	MPTP-induced mice	Reduce inflammation, autophagy, and apoptosis [75].
Perillyl alcohol	*Mentha haplocalyx*	200 µM	LPS and MPTP induced in vitro and in vivo study	Attenuates NLRP3 inflammasome activation and rescues dopaminergic neurons [76].
**Alkaloids**				
2-(Quinoline-8-carboxamido) benzoic acid	*Aspergillus* sp.	Various dose	MPP+-induced *Caenorhabditis elegans*	Modulates the formation of neurotoxic α-synuclein ameliorated induced dopaminergic neurodegeneration [77].
Melodicochine A	Stems and leaves of *Melodinus cochinchinensis*	0.72 to 17.89 μM	6-hydroxydopamine-induced SH-SY5Y cells	Neuroprotection [78].
Berberine	*Coptis chinensis* and *Berberis vulgaris*	100–200 mg/kg	Mice and bacteria	Improves brain dopa/dopamine levels [79].
Caffeine (1,3,7-trimethylxanthin)	Seeds and leaves of coffee (*Coffea arabica*), tea (*Camellia sinensis*) and cocoa (*Theobroma cacao* L.)	20 mg/kg (varied dose)	In various in vitro and in vivo models	Exhibits antioxidant properties and inhibits lipid peroxidation [80].
Lycopodium	Lycopodiaceae plants	50 mg/kg	Rotenone-induced rat	Reduction in pro-inflammatory response and α-synuclein expression. Also, synergistically enhances antioxidant defense via multimodal action [81].
